# Supplementary material for: Tunable rectification in a molecular heterojunction with two-dimensional semiconductors
Source: Nat Commun. 2020 Mar 16;11:1412. doi: 10.1038/s41467-020-15144-9 (PMC7075907; doi:10.1038/s41467-020-15144-9)
Supplement: Supplementary file 1 — Supplementary Information [file 41467_2020_15144_MOESM1_ESM.pdf]

Supplementary Information

Tunable rectification in a molecular heterojunction with two-dimensional semiconductors

Jaeho Shin[1,†], Seunghoon Yang[1,†], Yeonsik Jang[2], Jung Sun Eo[1], Tae-Wook Kim[3], Takhee Lee[2], Chul-Ho Lee[1,*], and Gunuk Wang[*1,*]

[1]KU-KIST Graduate School of Converging Science and Technology, Korea University, Seoul 02841, Korea

[2]Department of Physics and Astronomy, and Institute of Applied Physics, Seoul National University, Seoul 08826, Korea

[3]Functional Composite Materials Research Center, Institute of Advanced Composite Materials, Korea Institute of Science and Technology, Jeollabuk-do 55324, Korea

[*]Corresponding authors: gunukwang@korea.ac.kr and chlee80@korea.ac.kr

[†]These authors contributed equally to this work.

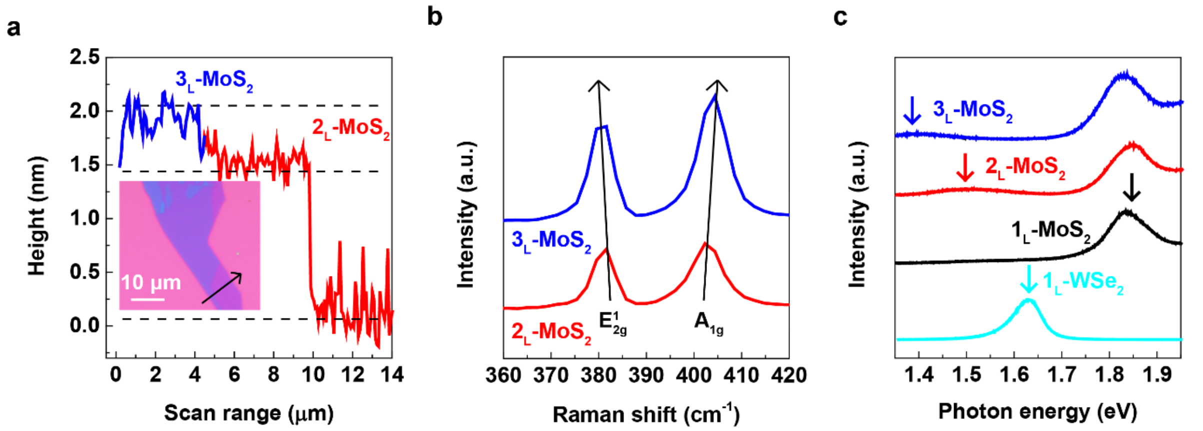

Supplementary Figure 1. Characterization of N_L-MoS$_2$ and 1_L-WSe$_2$ a, Topological line-profile of 2_L-MoS$_2$ (red line) and 3_L-MoS$_2$ (blue line) on SiO$_2$/Si substrate, investigated in non-contact AFM scanning mode. Black arrow in optical image (inset) indicates the investigation range of the line-profile. **b**, Raman spectra of 2_L-MoS$_2$ and 3_L-MoS$_2$. Two distinctive peaks corresponding to the E^1_{2g} and A_{1g} vibrational modes are observed at 381 cm^{-1} and 402 cm^{-1} in the profile of 2_L-MoS$_2$ and at 380 cm^{-1} and 404 cm^{-1} for 3_L-MoS$_2$. **c**, Photoluminescence spectra of N_L-MoS$_2$ and 1_L-WSe$_2$. The arrows indicate the strong excitonic emission peaks of N_L-MoS$_2$ and 1_L-WSe$_2$.[1,2]

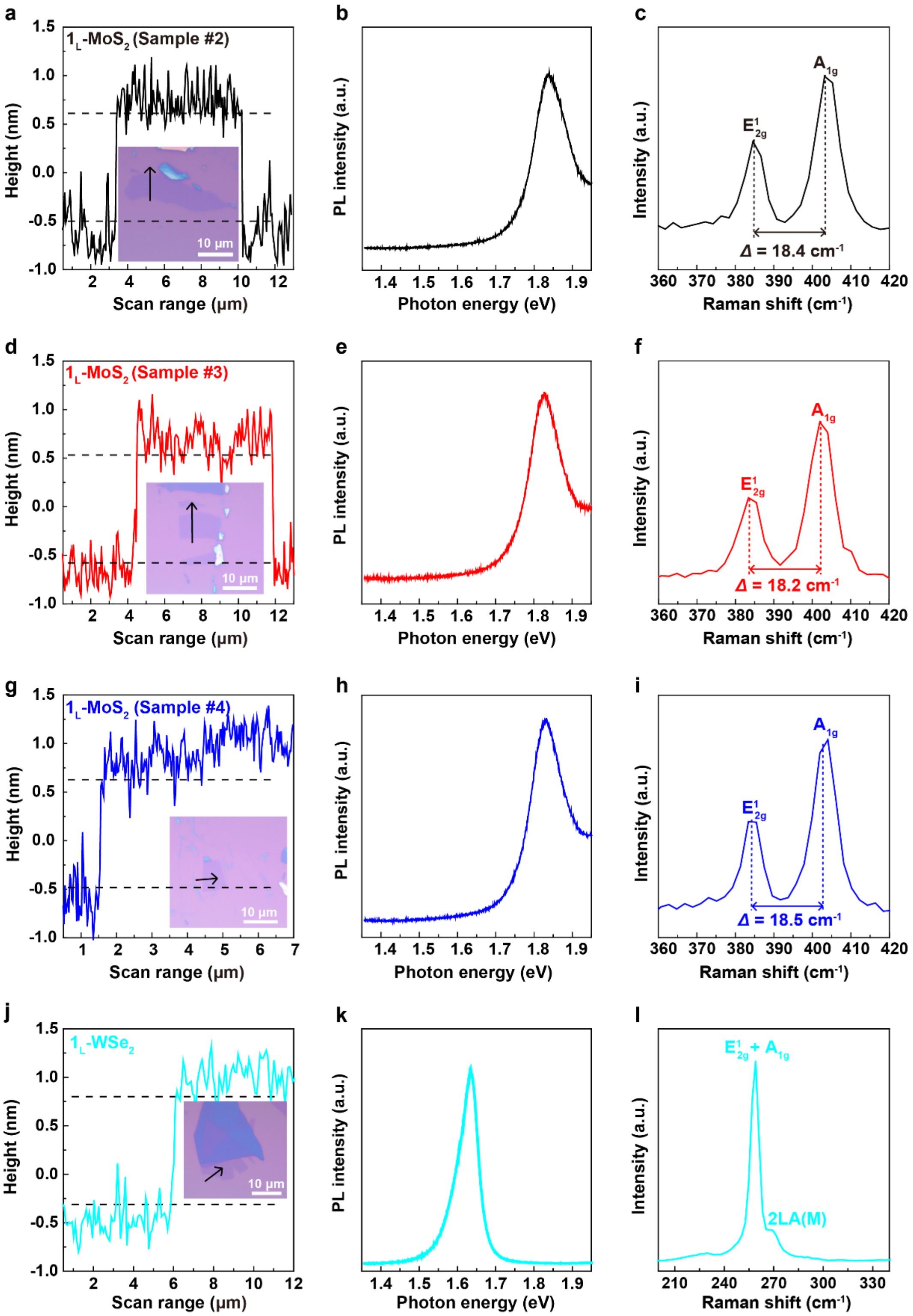

Supplementary Figure 2. Characterizations of different 1_L-MoS_2 and 1_L-WSe_2 samples (a, d, g, j), Topological line-profiles of different 1_L-MoS_2 samples (#2 (black), #3 (red), and #4 (blue)) and 1_L-WSe_2 (cyan) on SiO_2/Si substrate, investigated in non-contact AFM scanning

mode. Black arrows in optical image (inset) indicate the investigation range of the line profiles, respectively. **(b, e, h, k)**, Raman spectra of 1_L-MoS_2 samples (#2 (black), #3 (red), and #4 (blue)) and 1_L-WSe_2 (cyan). **(c, f, i, l)**, Photoluminescence spectra of 1_L-MoS_2 samples (#2 (black), #3 (red), and #4 (blue)) and 1L-WSe_2 (cyan).

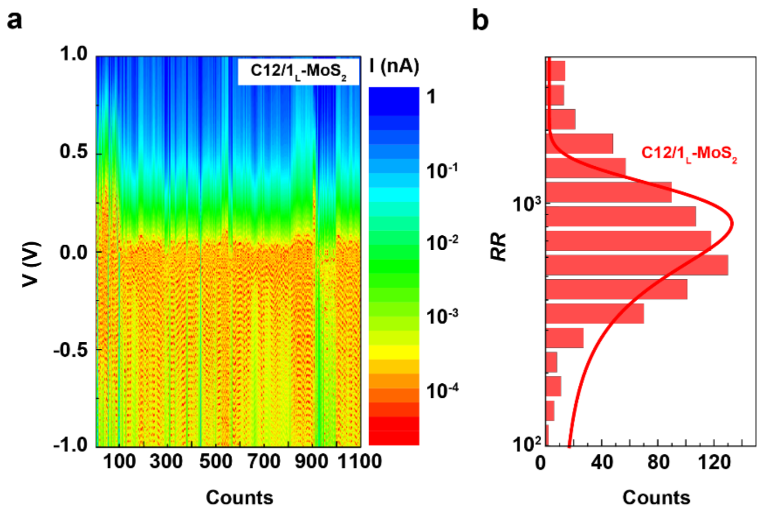

Supplementary Figure 3. Electrical characteristics of Au/C12/1_L-MoS_2/Au junction a, Contour maps of transport *I* for C12/1_L-MoS_2 junctions according to *V* and the number of junctions. **b**, Statistical histogram of *RR* for C12/1_L-MoS_2 junction. The line curves are fitting results from Gaussian function. Note the total number of C12/1_L-MoS_2 junctions is 1,100.

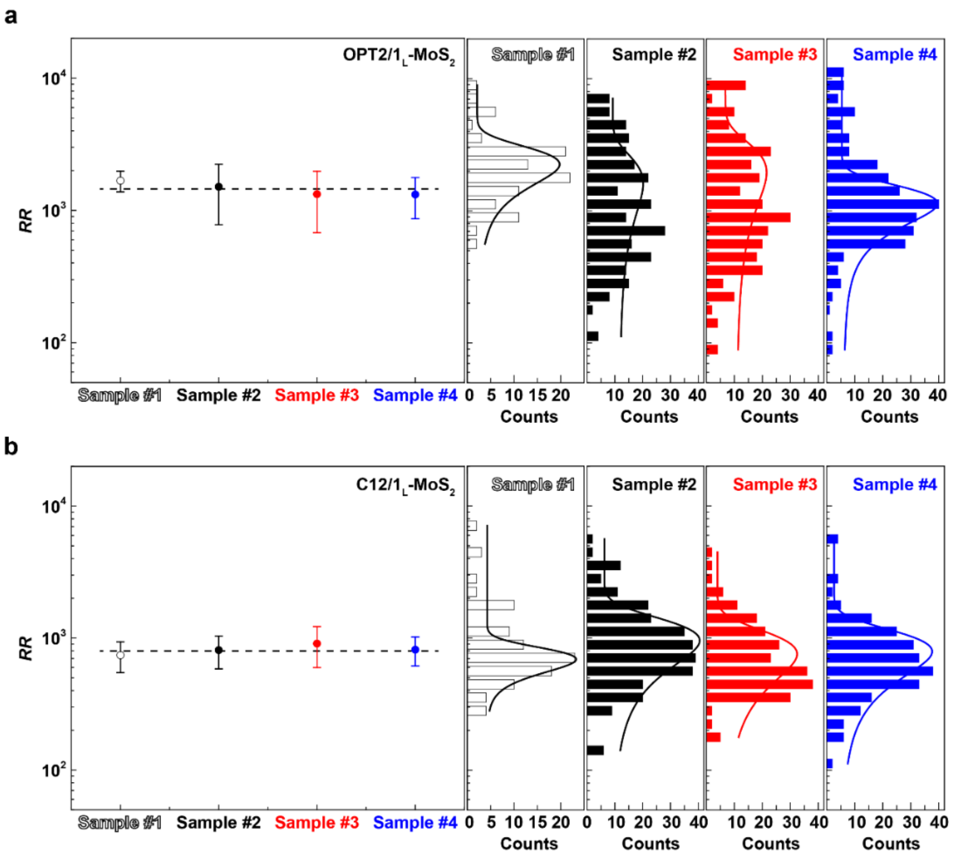

Supplementary Figure 4. Reproducibility of Au/OPT2 (C12)/1$_L$-MoS$_2$/Au junctions (a, b), *RR* plots of OPT2/1$_L$-MoS$_2$ and C12/1$_L$-MoS$_2$ junctions as a function of the sample numbers (#1-#4). Right graphs of **a** and **b** show the statistical histograms of *RR* for OPT2/1$_L$-MoS$_2$ junctions according to the sample numbers (#1-#4).

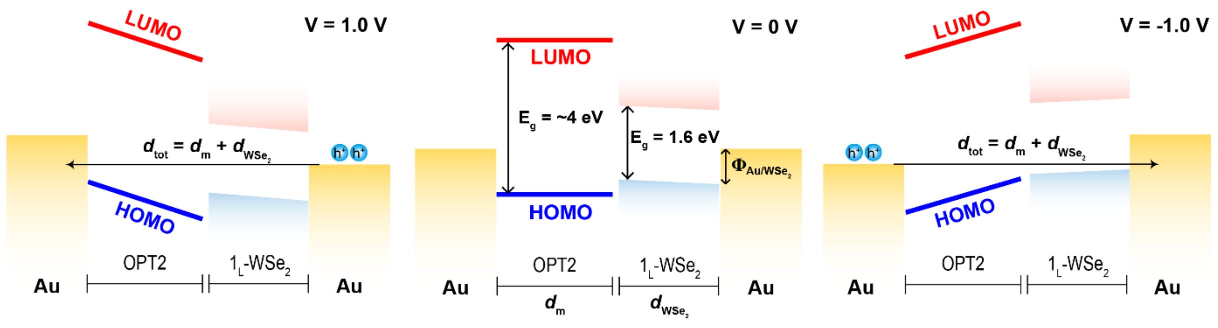

Supplementary Figure 5. Energy band alignment of Au/OPT2/1_L-WSe_2/Au junction Energy band alignment of Au/OPT2/1_L-WSe_2/Au junction at $V = 0$ V, $V = 1.0$ V, and $V = -1.0$ V. Note that the E_g of 1_L-WSe_2 is set to ~1.6 eV.[2]

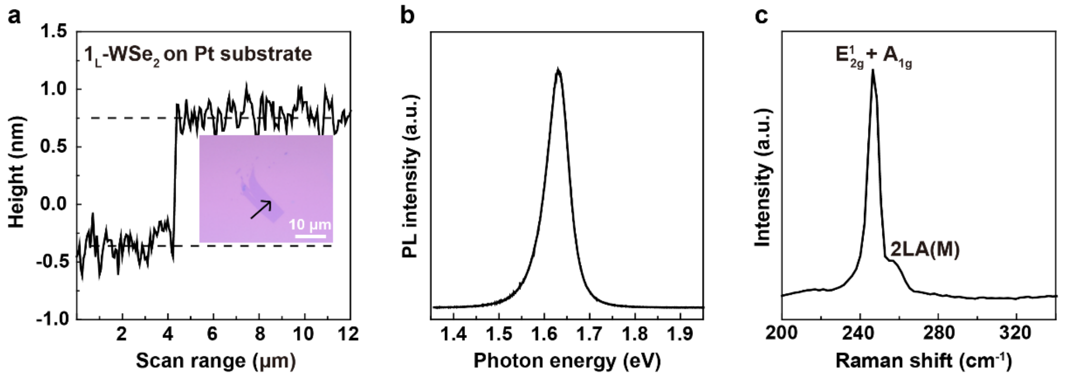

Supplementary Figure 6. Characterization of different 1_L-WSe_2 on Pt substrate a, Topological line-profile of 1_L-WSe_2 samples on Pt substrate, investigated by non-contact AFM scanning mode. Black arrow in optical image (inset) indicates the investigation range of the line profiles, respectively. **b**, Raman spectra of 1_L-WSe_2 samples. **c**, Photoluminescence spectra of 1_L-WSe_2 samples.

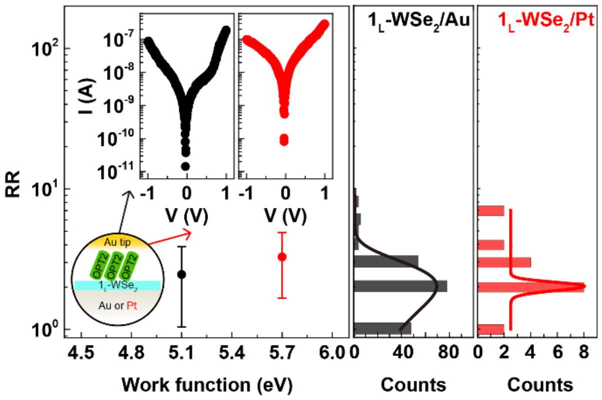

Supplementary Figure 7. Electrical characteristics of Au/OPT2/1_L-WSe$_2$/Au or Pt junctions *RR* plots of Au/OPT2/1_L-WSe$_2$/Au (black) and Au/OPT2/1_L-WSe$_2$/Pt (red), respectively (left). The statistical histograms of *RR* for Au/OPT2/1_L-WSe$_2$/Au (black) and Au/OPT2/1_L-WSe$_2$/Pt (red), respectively (right).

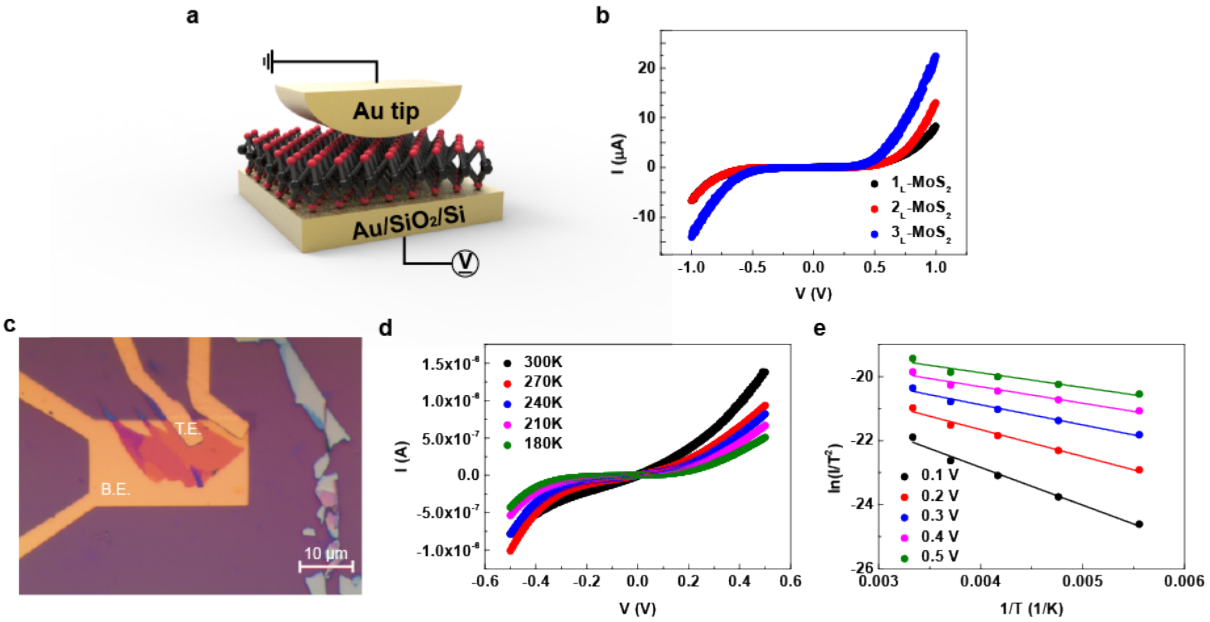

Supplementary Figure 8. Charge transport mechanism of Au/N_L-MoS_2/Au junction a, Schematic of Au/N_L-MoS_2/Au junction stacked on SiO_2/Si substrate using the CAFM technique. **b**, Representative *I-V* characteristics for Au/N_L-MoS_2 (N_L = 1_L (black), 2_L (red), and 3_L (blue))/Au junction at T = 300 K using the CAFM technique. Note that a large current is observed as the number of MoS_2 layers increases due to the reduction of the interfacial barrier height. **c**, Optical image of solid-state Au/Bulk-MoS_2/Au junction. **d**, *I-V* characteristics at temperatures from 180 to 300 K with a step of 30 K. **e**, Arrhenius plots generated from (**d**) at voltages from 0.1 to 0.5 V with 0.1 step. Note that the barrier height is extracted by applying the Schottky emission equation: $\ln\left(\frac{I}{T^2}\right) = -\frac{\Phi_{SB}}{kT} + \mathrm{c}$, where c is a constant and Φ_{SB} is the slope of the Arrhenius plot of $-1/kT$ vs. $\ln(I/T^2)$. The estimated Schottky barrier height changes from 101 eV to 39 eV as the applied voltage was increased from 0.1 V to 0.5 V.

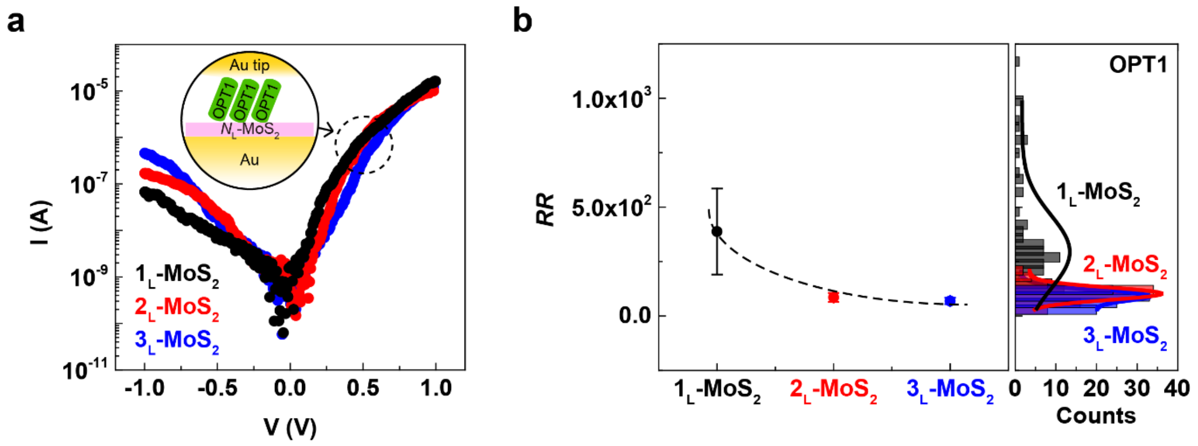

Supplementary Figure 9. Electrical characteristics of Au/OPT1/N_L-MoS_2/Au junctions a, Representative *I-V* characteristics of Au/OPT1/N_L-MoS_2 (N_L = 1_L, 2_L, or 3_L)/Au junction. **b,** *RR* plots of OPT1/N_L-MoS_2 junctions as a function of the number of MoS_2 layers. As the N_L is increased from 1_L to 3_L, the *RR* decreases from $(3.88 \pm 1.97) \times 10^2$ to $(6.89 \pm 1.49) \times 10^1$ for the OPT1/N_L-MoS_2 junctions. The statistical histogram of *RR* for the OPT1/N_L-MoS_2 junctions as a function of the number of MoS_2 layers are shown in the right figure. The error bars in **b** indicate the standard deviations of *RR* obtained from at least 100 different positions of each junction.

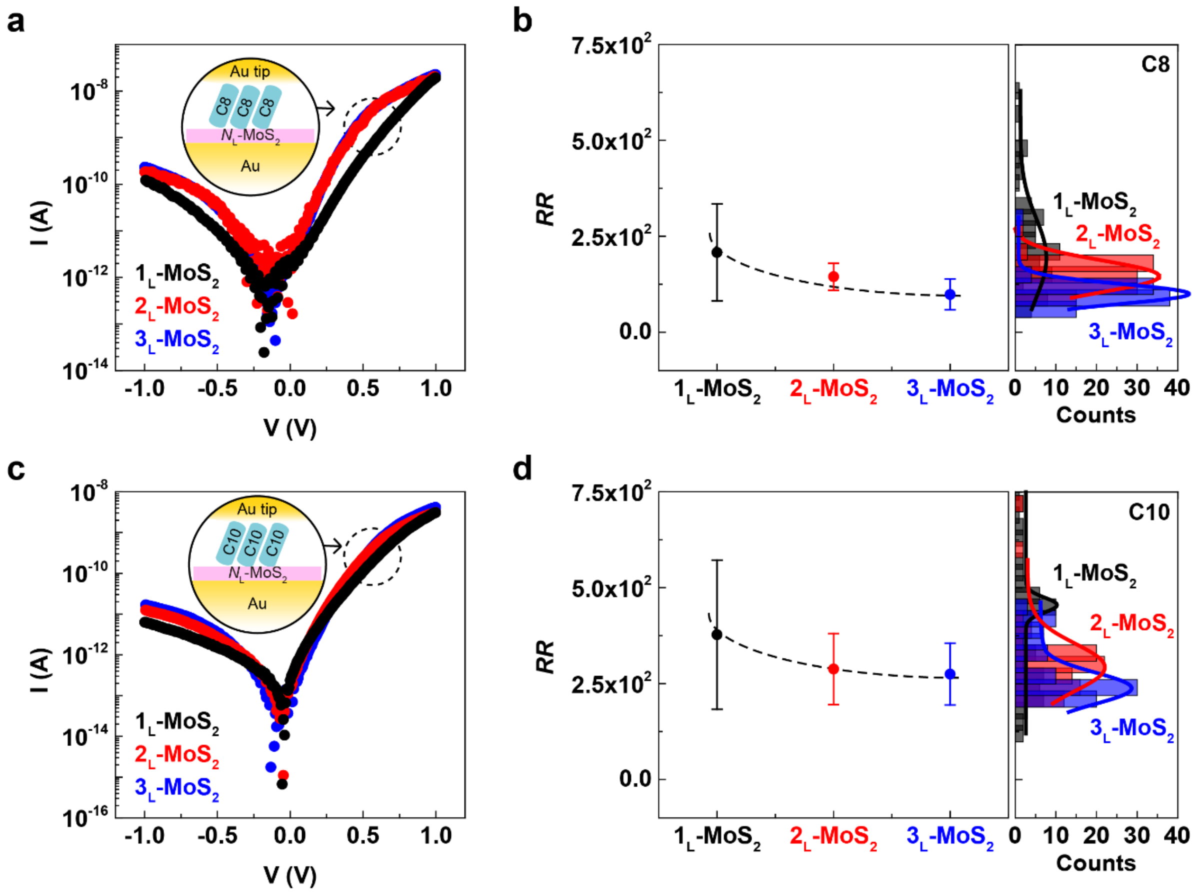

Supplementary Figure 10. Electrical characteristics of Au/C(n)/N_L-MoS$_2$/Au junctions a, Representative *I-V* characteristics of Au/C8/N_L-MoS$_2$ (N_L = 1_L, 2_L, or 3_L)/Au junction. **b,** *RR* plots of C8/N_L-MoS$_2$ junctions as a function of the number of MoS$_2$ layers. The *RR* decreases from (2.08 ± 1.26) × 10^2 to (9.84 ± 4.0) × 10^1 when the N_L is increased from 1_L to 3_L. **c,** Representative *I-V* characteristics of Au/C10/N_L-MoS$_2$ (N_L = 1_L, 2_L, or 3_L)/Au junction. **d,** *RR* plots of C10/N_L-MoS$_2$ junctions as a function of the number of MoS$_2$ layers. The *RR* decreases from (3.78 ± 1.94) × 10^2 to (2.75 ± 0.81) × 10^2 when the N_L is increased from 1_L to 3_L. The statistical histograms of *RR* for C8/N_L-MoS$_2$ and C10/N_L-MoS$_2$ junctions as a function of the number of MoS$_2$ layers are shown in the right figures (**b** and **d**).

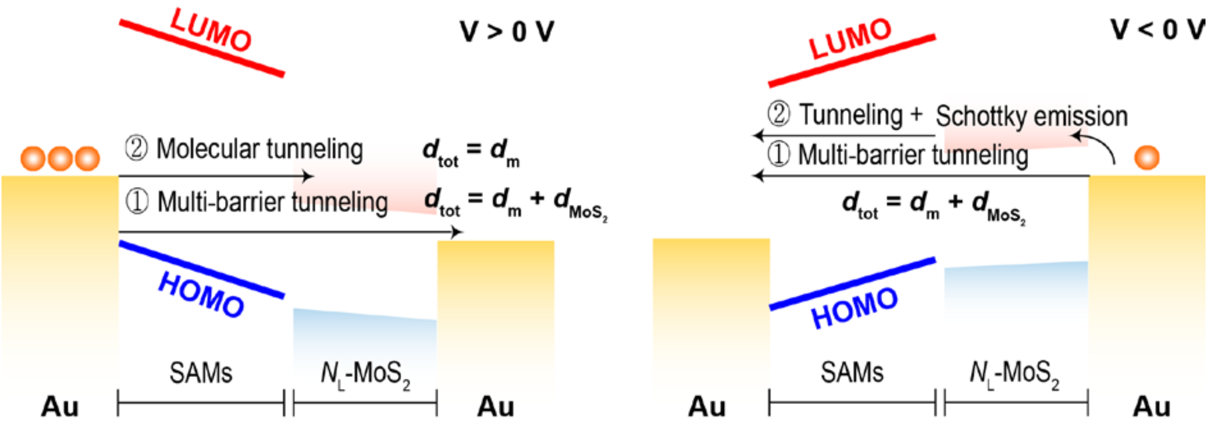

Supplementary Figure 11. Energy band alignment and possible charge transport pathways for the Au/SAMs/N_L-MoS_2/Au junction corresponding to $V > 0$ V and $V < 0$ V.

Pathway-dependent charge transport mechanism. The charge transport at relatively low positive voltage occurs sequentially in two tunnel barriers (i.e., molecular and N_L-MoS_2 barrier). Thus, the effective Φ could be extracted by applying the multi-barrier tunneling model:[3]

$$\Phi = \frac{\hbar}{2(2m)^{1/2}} \frac{\frac{2(2m)^{1/2}}{\hbar}(\Phi_m)^{1/2} d_m + \frac{2(2m)^{1/2}}{\hbar}(\Phi_{MoS2})^{1/2} d_{MoS2}}{d_m + d_{MoS2}} \qquad \text{Supplementary Equation (1)}$$

where d_m (d_{MoS2}) is the molecular length (N_L-MoS_2 thickness) and Φ_m (Φ_{MoS2}) is the molecular (N_L-MoS_2) barrier. As mentioned in relation to Figure 2d, because the Au-tip/SAM interface is chemically bonded by an Au-S covalent contact, the molecular orbital level could change in accordance with the shift in the E_F of the Au-tip.[4,5] Further, no chemical link can be formed between the molecular SAMs and TMD. Thus, the energy band alignment at the Au-tip/SAM and N_L-MoS_2/Au junctions could shift independently based on the applied voltage. Thus, Φ_{MoS2} can change according to the applied voltage, $\Phi_{MoS2}(V) = \Phi_{MoS2} - V_{applied}$. Based on this assumption, J could be extracted by applying the tunneling equation for transport pathway ① in Supplementary Figure 11 (left):[6]

$$J = \frac{q}{4\pi^2\hbar d_{tot}{}^2}\left[\left(\Phi - \frac{qV}{2}\right)\exp\left(-\frac{2d_{tot}\sqrt{2m}}{\hbar}\sqrt{\Phi - \frac{qV}{2}}\right) - \left(\Phi + \frac{qV}{2}\right)\exp\left(-\frac{2d_{tot}\sqrt{2m}}{\hbar}\sqrt{\Phi + \frac{qV}{2}}\right)\right] \quad \text{Supplementary Equation (2)}$$

where m is the mass of the majority carrier, $\hbar$ is Planck's constant, q is the electric charge, V is the applied voltage, and d_{tot} is the total tunneling width (in this case, $d_{\mathrm{tot}} = d_{\mathrm{m}} + d_{\mathrm{MoS2}}$). When the applied voltage is high enough to match the conduction band edge of N_{L}-MoS_2 in the applied bias window, the dominant charge transport mechanism is tunneling across the molecular tunnel barrier only. Thus, J could be calculated from Supplementary Equation (2), where $d_{tot} = d_m$ and $\Phi = \Phi_{\mathrm{m}}$ = LUMO level – E_{F} for transport pathway ② in Supplementary Figure 11 (left).

Similarly, at relatively low negative voltage, charge transport occurs sequentially across two tunnel barriers (i.e., the molecular and N_{L}-MoS_2 barriers) (i.e., transport pathway ① in Supplementary Figure 11 (right)), which can also be estimated from the multi-barrier tunneling model. However, Φ at $V < 0$ might be slightly different compared to that at $V > 0$. Because the E_{F} of the bottom Au electrode is strongly pinned near the conduction band edge of N_{L}-MoS_2,[7,8] the interfacial barrier height at N_{L}-MoS_2/SAMs does not change even when a voltage is applied. In this case, Φ_{m} can only change according to the applied voltage, $\Phi_{\mathrm{m}}(V) = \Phi_{\mathrm{m}} - V_{\mathrm{applied}}$. Based on this assumption, J could be extracted by applying the tunneling equation for transport pathway ① in Supplementary Figure 11 (right). At relatively high negative voltage, charge transport could occur by both tunneling and Schottky emission across each molecular and MoS_2 barrier (i.e., transport pathway ② in Supplementary Figure 11 (right)). In that case, the transport J can be extracted by applying the following equation:[9]

$$J = A^* \cdot T \exp\left[-\frac{q}{k_B T}\left(\Phi_{SB,eff} - \frac{V}{n}\right)\right] \cdot \exp\left(\beta \cdot d_m\right) \quad \text{Supplementary Equation (3)}$$

where A^* is the Richardson constant, T is the temperature, k_B is the Boltzmann constant, $\Phi_{SB,eff}$ is the effective Schottky barrier height between N_L-MoS_2/Au, n is the ideality factor of MoS_2, β is the attenuation factor for the molecules, and d_m is the molecular length. Note that the barrier height at the SAM/N_L-MoS_2 interface can be further increased with longer molecules due to the Fermi level unpinning effect. Based on this pathway-dependent charge transport mechanism according to the voltage polarity, the electrical characteristics and the *RR* for the molecular heterojunction systems were theoretically assessed (Figure 4 and Supplementary Figure 12).

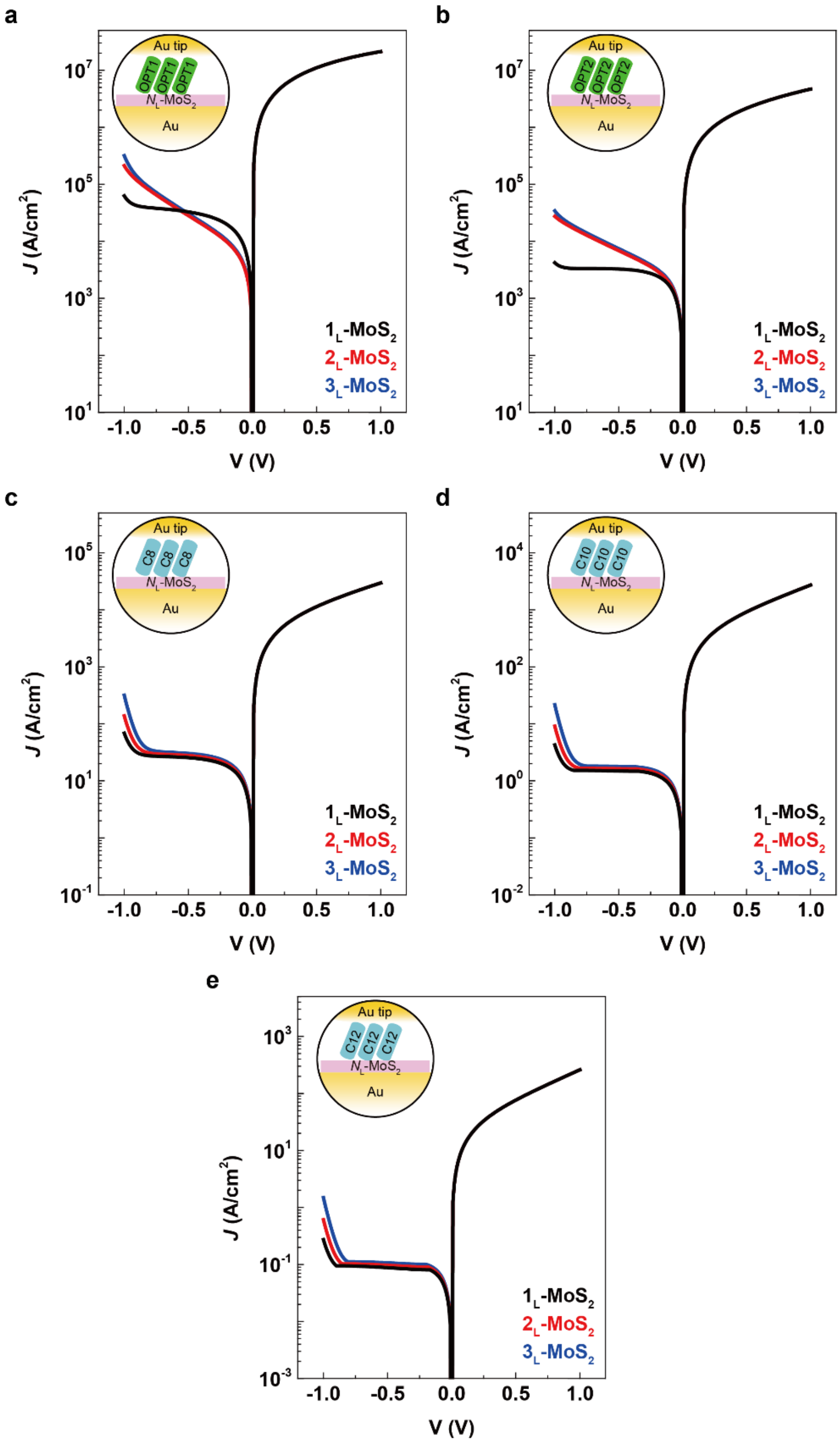

Supplementary Figure 12. Calculated *J-V* characteristics for the OPT(n)/N_L-MoS_2 and C(n)/N_L-MoS_2 junctions *J-V* plots for **a**, OPT1/N_L-MoS_2, **b**, OPT2/N_L-MoS_2, **c**, C8/N_L-MoS_2, **d**, C10/N_L-MoS_2, and **e**, C12/N_L-MoS_2 junctions, calculated from the pathway-dependent charge transport model.

Hertzian elastic-contact model

The Hertzian elastic contact model can be utilized to estimate the contact radius (a) between the Au tip and SAMs, $a = (RP_n/K)^{1/3}$, where R is the Au-tip radius (R = ~30 nm, Supplementary Fig. 13), P_n is the net force determined by F_L (tip loading force) + $F_{adhesion}$ (adhesion force), and K is elastic modulation (~20 GPa).[10] Note that it was found $F_{adhesion}$ = ~31.6 nN, 33.6 nN, ~13.2 nN, 15.4 nN, and 16.2 nN for OPT1, OPT2, C8, C10, and C12, respectively.[5,10] Based on these parameters, the contact radius, contact area, and the number of each molecular SAMs are estimated and summarized in Supplementary Table 1.

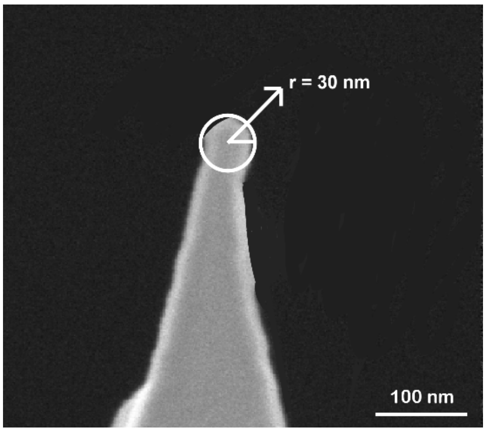

Supplementary Figure 13. SEM image of a CAFM Au probe tip.

	F_L (nN)	P_n (nN)	a (nm)	Contact area (nm^2)	N_o (nm^{-2})	# of molecules
**OPT1**	1	31.6	3.62	41.14	4.4	181
**OPT2**	1	34.6	3.73	43.71	4.4	192
**C8**	1	14.2	2.77	24.14	4.65	112
**C10**	1	16.4	2.91	26.57	4.65	123
**C12**	1	17.2	2.96	27.43	4.65	127

Supplementary Table 1. Summary for P_n, a, contact area, N_o, and the # of molecules of each molecular SAMs.

Supplementary references

1 Zhang, X. *et al.* Phonon and Raman scattering of two-dimensional transition metal dichalcogenides from monolayer, multilayer to bulk material. *Chem. Soc. Rev.* **44**, 2757-2785 (2015).

2 Tonndorf, P. *et al.* Photoluminescence emission and Raman response of monolayer MoS_2, $MoSe_2$, and WSe_2. *Opt. Express* **21**, 4908-4916 (2013).

3 Wang, G. *et al.* Electrical conduction through self-assembled monolayers in molecular junctions: Au/molecules/Au versus Au/molecule/PEDOT:PSS/Au. *Thin Solid Film* **518**, 824-828 (2009).

4 Wang, G., Kim, T.-W., Jang, Y. H. & Lee, T. Effects of metal−molecule contact and molecular structure on molecular electronic conduction in nonresonant tunneling regime: Alkyl versus conjugated molecules. *J. Phys. Chem. C* **112**, 13010-13016 (2008).

5 Shin, J. *et al.* Correlational effects of the molecular-tilt configuration and the intermolecular van der Waals interaction on the charge transport in the molecular junction. *Nano Lett.* **18**, 4322-4330 (2018).

6 Beebe, J. M., Kim, B., Gadzuk, J. W., Frisbie, C. D. & Kushmerick, J. G. Transition from direct tunneling to field emission in metal-molecule-metal junctions. *Phys. Rev. Lett.* **97**, 026801 (2006).

7 Gong, C., Colombo, L., Wallace, R. M. & Cho, K. The unusual mechanism of partial fermi level pinning at metal–MoS_2 interfaces. *Nano Lett.* **14**, 1714-1720 (2014).

8 Kim, C. *et al.* Fermi level pinning at electrical metal contacts of monolayer molybdenum dichalcogenides. *ACS Nano* **11**, 1588-1596 (2017).

9 Chattopadhyay, P. & Daw, A. On the current transport mechanism in a metal—insulator—semiconductor (MIS) diode. *Solid State Electron.* **29**, 555-560 (1986).

10 Song, H., Lee, H. & Lee, T. Intermolecular chain-to-chain tunneling in metal−alkanethiol−metal junctions. *J. Am. Chem. Soc.* **129**, 3806-3807 (2007).
